# Functional PTGS2 polymorphism-based models as novel predictive markers in metastatic renal cell carcinoma patients receiving first-line sunitinib

**DOI:** 10.1038/srep41371

**Published:** 2017-01-24

**Authors:** Arancha Cebrián, Teresa Gómez del Pulgar, María José Méndez-Vidal, María Luisa Gonzálvez, Nuria Lainez, Daniel Castellano, Iciar García-Carbonero, Emilio Esteban, Maria Isabel Sáez, Rosa Villatoro, Cristina Suárez, Alfredo Carrato, Javier Munárriz-Ferrándiz, Laura Basterrechea, Mirta García-Alonso, José Luis González-Larriba, Begoña Perez-Valderrama, Josefina Cruz-Jurado, Aránzazu González del Alba, Fernando Moreno, Gaspar Reynés, María Rodríguez-Remírez, Valentina Boni, Ignacio Mahillo-Fernández, Yolanda Martin, Andrea Viqueira, Jesús García-Foncillas

**Affiliations:** 1 Fundación Jiménez Díaz University Hospital, Madrid, Spain; 2 Reina Sofía University Hospital, Córdoba, Spain; 3 Virgen de la Arrixaca University Hospital, Murcia, Spain; 4 Complejo Hospitalario de Navarra, Pamplona, Spain; 5 12 de Octubre University Hospital, Madrid, Spain; 6 Virgen de la Salud University Hospital, Toledo, Spain; 7 Central University Hospital of Asturias, Oviedo, Spain; 8 Virgen de la Victoria University Hospital, Málaga, Spain; 9 Hospital Costa del Sol, Marbella, Spain; 10 Vall d’Hebron University Hospital, Barcelona, Spain; 11 Ramon y Cajal University Hospital, Madrid, Spain; 12 Hospital Provincial de Castellón, Castellón, Spain; 13 Hospital de Donostia, San Sebastian, Spain; 14 Insular de Gran Canaria University Hospital, Islas Canarias, Spain; 15 Clinico San Carlos University Hospital, Madrid, Spain; 16 Virgen del Rocío University Hospital, Sevilla, Spain; 17 Canarias University Hospital, Santa Cruz de Tenerife, Islas Canarias, Spain; 18 Son Espases University Hospital, Palma de Mallorca, Spain; 19 Fuenlabrada University Hospital, Fuenlabrada, Spain; 20 La Fe University Hospital, Valencia, Spain; 21 Center for Applied Medical Research, Pamplona, Spain; 22 Trial Form Support, Madrid, Spain; 23 Pfizer Oncology, Madrid, Spain

## Abstract

Sunitinib is the currently standard treatment for metastatic renal cell carcinoma (mRCC). Multiple candidate predictive biomarkers for sunitinib response have been evaluated but none of them has been implemented in the clinic yet. The aim of this study was to analyze single nucleotide polymorphisms (SNPs) in genes linked to mode of action of sunitinib and immune response as biomarkers for mRCC. This is a multicenter, prospective and observational study involving 20 hospitals. Seventy-five mRCC patients treated with sunitinib as first line were used to assess the impact of 63 SNPs in 31 candidate genes on clinical outcome. rs2243250 (IL4) and rs5275 (*PTGS2*) were found to be significantly associated with shorter cancer-specific survival (CSS). Moreover, allele C (rs5275) was associated with higher *PTGS2* expression level confirming its functional role. Combination of rs5275 and rs7651265 or rs2243250 for progression free survival (PFS) or CSS, respectively, was a more valuable predictive biomarker remaining significant after correction for multiple testing. It is the first time that association of rs5275 with survival in mRCC patients is described. Two-SNP models containing this functional variant may serve as more predictive biomarkers for sunitinib and could suppose a clinically relevant tool to improve the mRCC patient management.

Renal cell carcinoma (RCC) is the most common type of kidney cancer worldwide^1^. Approximately 25% of patients will have metastatic disease at presentation^2^ and, despite an attempted curative surgery, around 20–30% of patients will recur^1^. Over the last years, the clarification of molecular mechanisms has transformed its management.

Sunitinib is the gold standard drug for the treatment of metastatic RCC (mRCC) as first- and second-line therapy. It is a tyrosine kinase receptor inhibitor affecting angiogenesis pathway since it blocks VEGF receptors-1,−2,−3 as well as platelet-derived growth factor receptors (PDGFR-α and –β), FLT-3, RET and c-Kit^3^. Although 50% of RCC patients treated with sunitinib show an objective response and 43% reach disease stabilization, 7% will suffer progressive disease at first evaluation^4^. The identification of biomarkers able to predict sunitinib sensitivity/resistance could avoid unnecessary costs and side effects, guiding alternative treatment decisions.

The mammalian target of rapamycin (mTOR) pathway plays a key role in cell growth regulation and angiogenesis and, it is activated downstream by activation of tyrosine kinase receptors, such as VEGFR through phosphoinositide 3-kinase (PI3K)/Akt pathway. It has been recently described that sunitinib completely abrogates PI3K/AKT/mTOR survival signaling^5^.

One of the hallmarks of cancer is the inflammatory microenvironment^6^. Recently, it has been correlated the persistence of chronic inflammation and reduced survival in advanced RCC patients^7^,^8^. A wide variety of pro- and anti-inflammatory mediators regulate immune response, among them interleukin-1β (IL-1β) and tumor necrosis factor-α (TNF-α) are potent pro-inflammatory cytokines involved in both cancer development and progression. On the contrary, IL-4, IL-10 and tumor growth factor-β (TGF-β) are effective negative regulators of the immune response, and they have been involved at different levels in RCC disease such as cellular senescence^9^ and increased incidence of metastasis^10^,^11^.

In addition to cytokines and growth factors, cyclooxygenase 2 (COX-2) has been implicated in carcinogenesis and metastatic progression of different types of cancers including RCC^12^. COX-2 expression significantly correlates with increased microvessel density, higher stage and grade^13^,^14^. The gene which encodes COX-2 (*PTGS2*) is induced in response to cytokines, and other inflammatory and mitogenic stimuli.

Patient genetic background could play an important role especially with regard to the drugs that interact with the tumor microenvironment and non-malignant endothelial cells such as sunitinib. Genes involved in the pathways mentioned above harbor polymorphic variants that have been associated with the outcome in different cancers. However, little is known about their roles in mRCC, except for some located in angiogenesis-related genes. Trying to address this issue, we aimed to assess the predictive role of 63 single nucleotide polymorphisms (SNPs) in genes that affect the mode of action of sunitinib and inflammatory response.

## Results

A total of 75 mRCC patients receiving first-line sunitinib treatment was studied (Table 1). Median age at diagnosis was 63 years (range 26–87). According to the MSKCC prognostic criteria, patients were categorized into favourable (2%), intermediate (68%) and poor (23%) groups. At the time of analysis, 45 out of 75 patients (60%) had disease progression and 27 (36%) had died. The median follow up of the 75 patients was 12.2 months (range 1–28).

### Association of Genetic variants with Outcome

The genotypes and allele frequencies for each polymorphism are given in Table 2. The association of individual SNPs with PFS and CSS was assessed by Cox regression analysis under codominant, dominant, recessive and additive models and the best model was chosen (Supplementary Table S1). All those meeting a *P* < 0.1 were selected as candidate for multivariate Cox regression analysis (Table 3). Clinical and biochemical parameters associated with PFS and CSS were also assessed (Supplementary Table S2). To analyze the effect of SNPs on PFS and CSS, both the MSKCC score and prior nephrectomy were considered as covariates whereas histology was only considered for CSS.

Multivariate analyses showed that PFS was significantly associated with SNPs rs7651265 in *PIK3CA* and rs307826 in *FLT4* (Table 3). Cancer-specific survival was significantly associated with SNPs rs2243250 in *IL4* and rs5275 in *PTGS2*, both of them remained significant after correction for multiple testing using the Benjamini-Hochberg method (Table 3).

**Figure 1:**
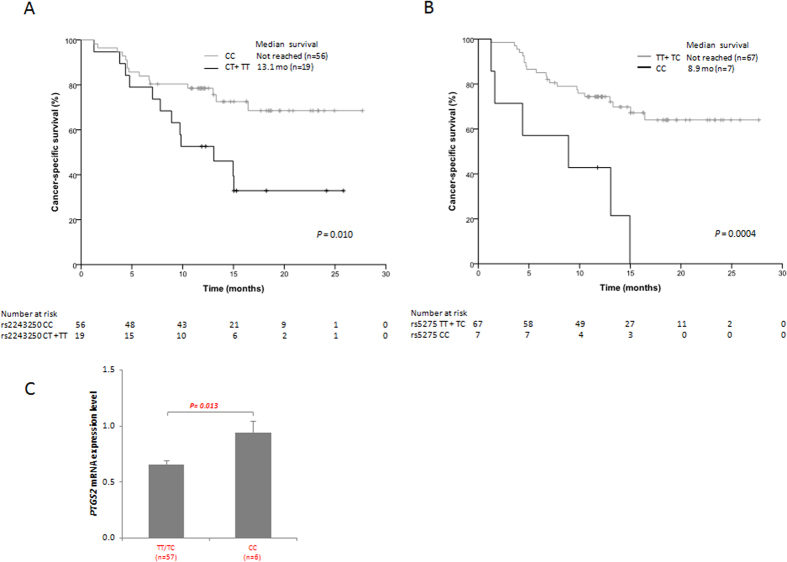
Association of single nucleotide polymorphism rs2243250 (*IL4*) (**A**) and rs5275 (*PTGS2*) (**B**) with cancer-specific survival in metastatic renal cell carcinoma patients treated with sunitinib. Correlation between *PTGS2* mRNA expression levels according to rs5275 genotypes (**C**). Gene expression was determined in patients carrying T allele (TT/TC) or homozygous for C allele. There is a significant difference between the T carriers and CC genotype (P = 0.013).

Specifically, the CT and TT genotypes for rs2243250 showed inferior median CSS compared with the wild-type CC genotype (13.1 months *vs.* not reached; HR, 4.69; 95% CI, 1.92–11.44; *P* = 0.0009; Table 3 and Fig. 1A). This SNP is located in the *IL4* promoter region and its functionality has been previously described^15^,^16^. Our result is in accordance with the prognostic value of this variant in immunotherapy-treated mRCC patients^17^.

Regarding to rs5275 in *PTGS2*, a total of 67 patients had TT/TC genotypes, whereas 7 patients were CC. Median CSS was not reached for TT/TC patients, and was only 8.9 months for rare homozygous patients (HR, 5.22; 95% CI, 1.70–15.98; *P* = 0.01; Table 3 and Fig. 1B). As this SNP is located in the 3’ untranslated region (3’UTR) of *PTGS2* mRNA and disrupts microRNA-mediated regulation^18^, *PTGS2* mRNA expression levels were compared between the two groups of patients (TT/TC vs CC). We confirmed that CC patients showed higher levels of *PTGS2* expression than TT/TC patients (Fig. 1C).

### Predictive two-SNPs models for outcome

Among the significant SNPs after multivariate adjustment for PFS or CSS (Table 3), we tested different combinations in order to find models with higher predictive value than the single predictors. To assess and compare the predictive ability of the individual variants and the two-SNPs models, the AUC was determined.

**Figure 2:**
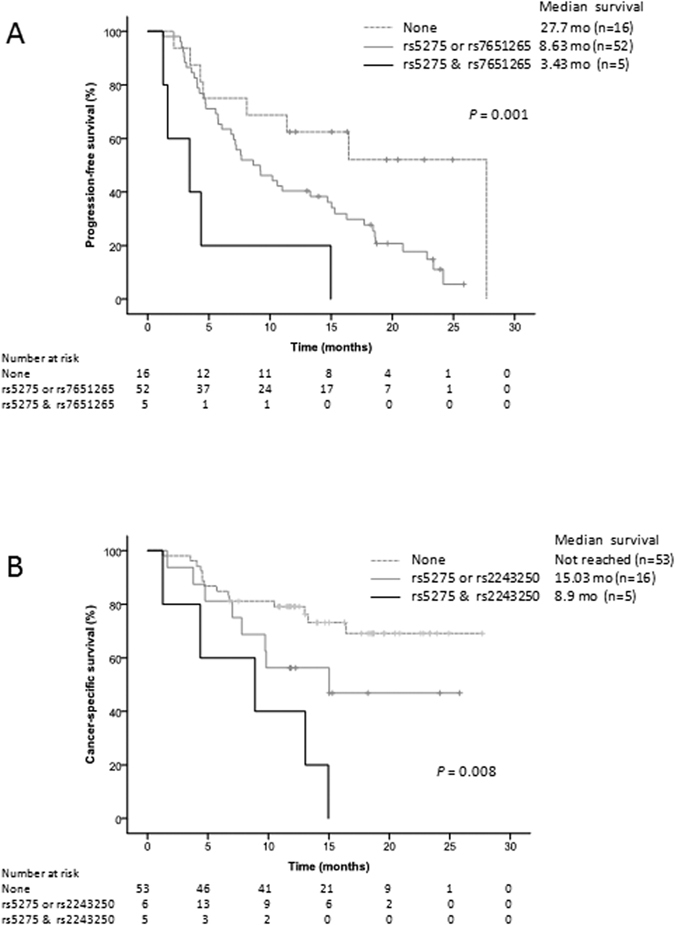
Kaplan-Meier curves for each of the proposed two-SNPs models. (**A**) Progression-free survival for patients grouped by adverse genotypes in rs5275 (*PTGS2)* and rs7651265 (*PIK3CA)*. (**B**) Cancer-specific survival for patients stratified according to combination of deleterious genotypes in rs5275 (*PTGS2)* and rs2243250 (*IL4*).

Three different combinations were tested for PFS (rs307826 and rs7651265; rs5275 and rs7651265; rs307826 and rs5275). We found that the combination of deleterious genotypes for rs5275 (CC) in *PTGS2* and rs7651265 (AA) in *PIK3CA* was strongly associated with lower PFS (*P* = 0.001; Fig. 2A). As shown in Table 4, patients with both deleterious genotypes were almost 5.5 times more likely to progress compared to those without these genotypes (HR, 5.44; 95% CI, 1.39–21.32; *P* = 0.015). The predictive capacity of this model was better than the individual SNPs since its AUC was 0.702 compared to 0.595 (rs5275) and 0.671 (rs7651265) (Supplementary Table S3). This combination was the best predictor to discriminate the three risk groups compared to the other two combinations (rs307826 and rs7651265; rs307826 and rs5275) (Supplementary Table S4). Regarding to CSS, three risk groups were identified according to the deleterious genotypes combination of polymorphisms rs5275 (CC) in *PTGS2* and rs2243250 (CT/TT) in *IL4 (P* = 0.008; Fig. 2B). This two-SNPs model was strongly associated with a higher risk of death (HR, 7.32; 95% CI, 2.10–25.54; *P* = 0.002; Table 4) and showed higher ability to predict CSS (AUC = 0.671) than each SNP separately (AUC = 0.649) (Supplementary Table S3).

Both combination of *PTGS2* & *PIK3CA* for PFS and combination of *PTGS2* & *IL4* for CSS remained significant after multiple testing correction.

## Discussion

A set of polymorphisms located in genes involved in the mode of action of sunitinib and in the immune response were assessed as potential markers of mRCC outcome.

IL-4 is a pleiotropic cytokine which mediates a variety of interactions among components of the immune system. High levels of IL-4 in the tumour microenvironment have been correlated with the grade of malignancy and tumor resistance to apoptosis^19^. The functional genetic variant, rs2243250, located at the promoter region, drives more than 3-fold greater *IL4* expression when T allele is present since allele T showed greater binding to nuclear transcription factors than allele C^15^. Nakashima *et al*. assessed the functional consequences of this SNP *in vivo* and found that the mean percentages of peripheral Th cells for patients with genotype CC was significantly lower than those with allele T (TT/TC)^16^. Little is known about the role of this variant in cancer since few studies have analyzed this SNP in the context of cancer risk and, only a single-center study in immunotherapy-treated mRCC patients has described it as genetic prognostic factor^17^. In this study, we show the significant association of rs2243250 with the sunitinib response since mRCC patients with T-allele have shorter cancer-specific survival. The fact that significance between genotypes with or without T allele has been observed in the proportion of Th cells producing IL4^16^ makes it likely that IL4 genotype could influence the type of immune response. Therefore, it might be related to the prevention of effective immune surveillance by TH1 cells during disease progression^17^.

Moreover, we demonstrate clearly, for the first time, the predictive role of the functional SNP rs5275 in *PTGS2* gene in mRCC patients. T-allele conferred a significant survival advantage since the rare homozygous variant is associated with more than five-fold increased risk of cancer-related death. This finding is consistent with the observed effect in early breast cancer and in advanced colorectal cancer where rs5275 was correlated with distant disease-free survival^20^ and, with progression-free and overall survival, respectively^21^. It is noteworthy that rs5275 improves the potential risk prediction found in other different genetic markers in similar series of mRCC treated with sunitinib^22^.

The genetic variant rs5275 is located in the 3’UTR of the *PTGS2* gene that encodes the protein COX2. It has been described that C allele disrupts miR-542–3p binding, allowing for mRNA stabilization and leading to a significant increase in both COX2 mRNA and protein levels in colorectal cancer^18^. Our results confirm the association of C allele in mRCC patients with higher mRNA expression levels, thereby expecting an increase in protein level. This presumption is supported by the correlation of high COX2 protein levels with shorter progression-free and cancer-specific survival in clear cell RCC^23^ and it is in agreement with the known role of COX2 in cancer^24^.

In order to evaluate the cumulative effect of the most relevant SNPs, different combinations have been tested. For PFS, a combination of rs5275 (*PTGS2*) and rs7651265 (*PIK3CA*) resulted in a promising marker for determining risk of progression after sunitinib treatment. A difference of 24 months in survival between high and low risk groups of patients was observed and remained significant after the Benjamini-Hochberg correction for multiple comparisons (Table 4). So far, rs7651265 (*PIK3CA*) has been only associated with susceptibility to ovarian and colon cancer^25^,^26^. Therefore, this is the first association with cancer patient outcome. The role of this biomarker in mRCC is supported by the recent study performed by the Cancer Genome Atlas Research Network confirming the PI3/AKT pathway as one of the most relevant pathways in RCC^27^.

In the case of CSS, the combination of the deleterious genotypes for rs5275 (*PTGS2*) and rs2243250 *(IL4)* showed more than 7 times higher death risk compared to the patients with none of deleterious genotypes. This model provided a better predictive capacity than each SNP individually (Supplementary Table S3).

Interestingly, the two-SNPs models associated with PFS and CSS harboured the variant rs5275 in *PTGS2* supporting for the first time the relevance of this functional genetic variant as predictive marker in mRCC.

One of the proposed mechanisms of resistance to anti-angiogenic therapy is that in the presence of agents targeting the VEGF pathway, alternative pro-angiogenic pathways are activated. The identification of predictive genetic markers in *PTGS2, IL4* and *PIK3CA* highlights, for the first time, the relevance of tumour microenvironment, inflammatory response and PI3K/AKT pathway activation in sunitinib treatment resistance. The functionality of the variants in *IL4* and *PTGS2* which leads to increased mRNA levels and thereby to higher expression of IL-4 and COX2 is associated with a lower potential clinical benefit of sunitinib.

The strength of these proposed predictors is that they are detected in the germline DNA, which is inherited, and insensitive to time and environmental factors. Since no reliable cancer predictive biomarker for sunitinib response has been implemented in the clinic yet, validation of our findings in an independent series of mRCC patients is warranted before put these markers into practice.

Our study had limitations. Schedule and dose modifications were not dictated by central protocol, and timing for radiological assessments was done according to each institution’s policy. Thus, courses of treatment were not standardized for the study and outcomes were assessed with regard to present practice. Finally, our study did not include a prospective, external validation. Because our patients were Caucasian, the relevance of these polymorphisms needs to be assessed in other ethnic groups. On the other hand, the SNPs that we found to be associated with sunitinib outcome are relatively common (13% for rs7651265, 13% for rs2243250, 16% for rs307826, and 28% for rs5275) and in addition, two of them (rs2243250 and rs5275) has been proven to be functional, which increases the power of the study. These factors are probably the major contributors to the robustness of our results, with statistically significant outcomes that persisted after adjustment for multiple testing. Our results warrant pharmacokinetic studies to better understand the molecular mechanisms and further validation in independent series.

The use of these genetic variants as novel predictive markers of response to sunitinib could provide a clinically relevant tool to improve mRCC patient management. In this way, if patients with a specific genotype are unlikely to benefit from sunitinib, an alternative therapy should then be used.

## Methods

### Patients

This is a multicenter, prospective and observational study in which patients with any subtype of mRCC and receiving any standard treatment were recruited. Twenty different hospitals enrolled patients between April 29, 2009, and July 15, 2010, and closed the follow-up database in March 20, 2012. This study was approved by the Institutional Ethical Committee at the Navarra Regional Government (authorization number 3066K1 4433) and the methods were carried out in accordance with the approved guidelines and regulations. All patients signed written informed consent before recruitment. Only patients treated with sunitinib as monotherapy in first line were analyzed since it was the most homogeneous subgroup. Drug treatment schedule and dose-reduction policy were decided by the attending doctors, in accordance with the current local practice guidelines and regulations. The inclusion criteria comprised patients that should be at least 18 years of age, with a verifiable diagnosis of mRCC. Patients who had been previously treated with any other medical therapy for RCC, and/or with history of other cancer disease apart RCC were excluded, except for those who had received curative treatment and were cancer-free in the last 5 years. Following the above mentioned criteria, eighty-two patients were assessed for eligibility.

According to the most widely used prognostic factor model from Memorial Sloan-Kettering Cancer Center (MSKCC)^28^, patients were categorized into favourable, intermediate and poor prognosis groups. The MSKCC score is based on 5 risk factors: low Karnofsky performance status ( < 70%), high lactate dehydrogenase levels ( > 1.5 times the upper limit of normal), low serum hemoglobin level, high corrected serum calcium concentration ( > 10 mg/dL), and time from initial diagnosis to treatment < 1 year.

### Genetic polymorphisms and genotyping

Genomic DNA was isolated from peripheral blood using the QIAamp DNA Mini Kit (Qiagen, Hilden, Germany), according to the manufacturer. Seven patients were excluded because of low DNA quality or poor DNA yield.

A total of 63 SNPs in 31 different genes were selected. The chosen SNPs were located in genes affecting sunitinib pharmacodynamics: PDGF- and VEGF-dependent angiogenesis (*ARNT, HIF1A, FLT4, KDR, PDGFRA, PGF* and *VEGFA)* or pro-angiogenic pathways (*CXCL12, FGFR2, FGFR4* and *IL8)*. Also were selected those genes encoding other sunitinib targets (*FLT3, RET*), involved in the PI3K/AKT pathway (*AKT1, AKT2, AKT3, PIK3CA, PTEN, NOS3*), or in the mTOR pathway (*MTOR, RHEB, RPTOR, RICTOR, TSC2*) and, a set of genes encoding cytokines and inflammatory mediators (*IL1B, IL4, IL10, PTGS2, TGFB1, TGFBR1 and TNF*).

The SNP genotyping was performed using Taqman products (Applied Biosystems, Foster City, CA, USA). The PCR was performed on the 7500 Real-Time PCR System (Applied Biosystems).

### *PTGS2* mRNA expression analysis

Total RNA was isolated from peripheral blood using the PAXgene Blood RNA Isolation System (PreAnalytiX GmbH, Switzerland). cDNA was synthesized using High Capacity cDNA Archive Kit (Applied Biosystems).

*PTGS2* gene expression was analyzed using 7500 Fast Real-Time PCR System (Applied Biosystems) and normalized to *PSMB4* expression. The probes used were Hs00153133_m1 for *PTGS2* and Hs00160598_m1 for *PSMB4*.

### Statistical analysis

Progression-free survival (PFS) was defined as the time between the first day of sunitinib and the date of progressive disease (PD) according to Response Evaluation Criteria in Solid Tumours (RECIST), clear clinical evidence of PD or death due to PD, or was censored at last follow-up. Cancer-specific survival (CSS) was defined as the time from the first day of sunitinib treatment and the date of death from cancer or was censored at the date of last follow-up.

Cox regression analysis was used to correlate each SNP with PFS and CSS. All genetics and clinical variables with a *P* < 0.1 were selected as candidate for multivariate Cox regression analysis. Survival curves were determined by the Kaplan-Meier method, with log-rank tests assessing the differences between the groups.

Predictive two-SNPs models for PFS or CSS were developed combining significant variants in the multivariate analyses. The predictive ability of the models was assessed using the area under the receiver operating characteristic (ROC) curve (AUC).

All individual and combined SNPs that were significant after multivariate analysis were corrected for multiple comparisons using Benjamini-Hochberg false discovery rate test. Statistical analyses were done using SPSS v20 software and R 3.1.0. *P* values ≤ 0.05 were considered statistically significant.

## Additional Information

**How to cite this article**: Cebrián, A. *et al*. Functional PTGS2 polymorphism-based models as novel predictive markers in metastatic renal cell carcinoma patients receiving first-line sunitinib. *Sci. Rep.* **7**, 41371; doi: 10.1038/srep41371 (2017).

**Publisher's note:** Springer Nature remains neutral with regard to jurisdictional claims in published maps and institutional affiliations.

## Supplementary Material

Supplementary Information

## Figures and Tables

**Table 1:** Patient characteristics.

	Patients (n = 75)
Age	
Median, y	63
Range	26–87
Gender	
Male	50 (67%)
Female	25 (33%)
Histology	
Clear cell	56 (75%)
Other	13 (17%)
Undetermined	6 (8%)
Prior nephrectomy	
No	16 (21%)
Yes	59 (79%)
Fuhrman grade	
G1-G2	14 (19%)
G3-G4	32 (42%)
Undetermined	29 (39%)
ECOG performance status	
0	28 (37%)
1	38 (51%)
2	9 (12%)
Number of metastatic sites	
< 2	33 (44%)
> 2	42 (56%)
Platelets	
< 400000	61 (81%)
> 400000	13 (17%)
Undetermined	1 (1%)
MSKCC score	
Favourable	2 (2%)
Intermediate	51 (68%)
Poor	17 (23%)
Undetermined	5 (7%)

Abbreviations: ECOG, Eastern Cooperative Oncology Group; MSKCC, Memorial Sloan-Kettering Cancer Center.

**Table 2:** Polymorphisms genotyped and allele frequency.

Gene	SNP	Variation	Patients*	Homozygous wild-type	Heterozygous	Homozygous variant	Observed MAF	MAF in dbSNP
*AKT1*	rs3803304 C > G	Intron	75	41	28	6	0.27	0.22
*AKT1*	rs2498804 G > T	3´near gene	75	31	35	9	0.35	0.34
*AKT1*	rs2494738 G > A	Intron	71	57	13	1	0.11	0.07
*AKT1*	rs1130214 G > T	5´-UTR	75	41	31	3	0.25	0.28
*AKT2*	rs8100018 G > C	Intron	75	43	29	3	0.23	0.26
*AKT2*	rs892119 G > A	Intron	75	55	19	1	0.14	0.14
*AKT3*	rs12045585 G > A	Intron	75	54	20	1	0.15	0.13
*AKT3*	rs2994329 G > A	Intron	74	46	27	1	0.20	0.20
*ARNT*	rs2228099 C > G	V189V	73	24	41	8	0.39	0.39
*CXCL12*	rs1801157 G > A	3´-UTR	73	41	32	0	0.22	0.21
*FGFR2*	rs2981582 C > T	Intron	75	32	24	19	0.41	0.46
*FGFR4*	rs351855 C > T	G388R	73	34	33	6	0.31	0.28
*FLT3*	rs1933437 T > C	T227M	73	21	40	12	0.44	0.34
*FLT4*	rs307826 A > G	T494A	74	53	19	2	0.16	0.10
*HIF1A*	rs11549465 C > T	P582S	72	53	17	2	0.15	0.07
*IL1b*	rs1143634 C > T	F105F	73	39	31	3	0.25	0.21
*IL4*	rs2243250 C > T	Promoter	75	56	18	1	0.13	0.14
*IL8*	rs4073 T > A	5´near gene	72	24	36	12	0.42	0.40
*IL10*	rs1800896 A > G	5´near gene	74	23	40	11	0.42	0.47
*IL10*	rs1800872 C > A	5´near gene	73	36	29	8	0.31	0.21
*KDR*	rs2305948 C > T	V297L	75	65	10	0	0.07	0.08
*KDR*	rs1870377 T > A	Q472H	74	43	28	3	0.23	0.27
*KDR*	rs2071559 C > T	5´near gene	74	22	30	22	0.50	0.49
*KDR*	rs7692791 T > C	Intron	74	18	41	15	0.48	0.48
*KDR*	rs1531289 C > T	Intron	74	32	34	8	0.34	0.29
*MTOR*	rs11121704 T > C	Intron	74	37	33	4	0.28	0.27
*MTOR*	rs2295080 T > G	3´near gene	74	31	34	9	0.35	0.30
*MTOR*	rs1074078 C > T	5´near gene	75	28	35	12	0.39	0.32
*NOS3*	rs1799983 G > T	D298E	72	22	37	13	0.44	0.34
*PDGFRA*	rs35597368 T > C	S478P	73	55	16	2	0.14	0.13
*PDGFRA*	rs1800813 G > A	5´near gene	74	46	25	3	0.21	0.21
*PDGFRA*	rs1800810 C > G	Promoter	73	45	24	4	0.22	0.21
*PDGFRA*	rs1800812 G > T	Promoter	74	43	25	6	0.25	0.21
*PGF*	rs8185 A > G	3´-UTR	74	52	20	2	0.16	0.17
*PIK3CA*	rs7651265 A > G	Intron	74	56	17	1	0.13	0.12
*PIK3CA*	rs7640662 C > G	Intron	75	54	19	2	0.15	0.14
*PIK3CA*	rs7621329 C > T	Intron	75	40	29	6	0.27	0.12
*PIK3CA*	rs6443624 C > A	Intron	75	36	29	10	0.33	0.17
*PIK3CA*	rs2699887 G > A	Intron	75	42	31	2	0.23	0.26
*PTEN*	rs2299939 C > A	Intron	75	55	17	3	0.15	0.20
*PTEN*	rs12569998 T > G	Intron	74	59	14	1	0.11	0.14
*PTEN*	rs12357281 G > C	Intron	75	66	9	0	0.06	0.06
*PTGS2*	rs5275 T > C	3´-UTR	74	40	27	7	0.28	0.38
*RET*	rs1799939 G > A	G691S	73	48	21	4	0.20	0.11
*RHEB*	rs717775 A > C	Intron	74	34	33	7	0.32	0.28
*RICTOR*	rs2043112 C > T	S837F	75	26	35	14	0.42	0.41
*RPTOR*	rs7211818 A > G	Intron	75	46	25	4	0.22	0.24
*RPTOR*	rs11653499 A > G	Intron	74	40	23	11	0.30	0.30
*RPTOR*	rs7212142 G > A	Intron	75	20	42	13	0.45	0.38
*RPTOR*	rs9674559 A > G	Intron	74	45	25	4	0.22	0.25
*TGFB1*	rs1800469 C > T	5´near gene	66	26	33	7	0.36	0.29
*TGFBR1*	rs868 A > G	3´-UTR	74	46	24	4	0.22	0.20
*TNF*	rs1800629 G > A	Intron	73	62	10	1	0.08	0.17
*TNF*	rs361525 G > A	5´near gene	74	63	1	10	0.14	0.07
*TNF*	rs1799724 C > T	5´near gene	74	61	13	0	0.09	0.07
*TSC2*	rs2073636 C > T	Intron	74	29	35	10	0.37	0.44
*TSC2*	rs8063461 G > A	Intron	75	28	39	8	0.37	0.44
*VEGFA*	rs2010963 G > C	5´-UTR	75	32	35	8	0.34	0.20
*VEGFA*	rs1570360 G > A	5´near gene	74	42	25	7	0.26	0.28
*VEGFA*	rs699947 C > A	5´near gene	75	26	35	14	0.42	0.48
*VEGFA*	rs3025039 C > T	3´-UTR	74	54	18	2	0.15	0.18
*VEGFA*	rs25648 C > T	S178S	74	54	17	3	0.16	0.18
*VEGFA*	rs2146323 C > A	Intron	74	37	30	7	0.30	0.34

Abbreviations: dbSNP, SNP database (http://www.ncbi.nlm.nih.gov/snp/); MAF, minor allele frequency; rs, reference SNP; SNP, single nucleotide polymorphism; UTR, untranslated region.

*Patients successfully genotyped.

**Table 3:** Univariate and multivariate analyses of polymorphisms associated with progression-free survival and cancer-specific survival in patients with metastatic renal cell carcinoma treated with sunitinib.

Gene	SNP	Inheritance model	Progression-free survival				Cancer-specific survival		
			*P* (Univariate)	*P* (Multivariate)	HR (95% CI)		*P* (Univariate)	*P* (Multivariate)	HR (95% CI)
*AKT1*	rs3803304	Additive	0.039	0.076	0.66 (0.42–1.06)		0.103	—	
*AKT1*	rs2494738	Dominant	0.530	—			0.058	0.078	2.73 (0.94–7.96)
*FLT4*	rs307826	Additive	0.038	0.038	1.42 (1.03–1.95)		0.863	—	
*IL4*	rs2243250	Dominant	0.224	—			0.016	0.0009*	4.69 (1.92–11.44)
*KDR*	rs1870377	Dominant	0.762	—			0.085	0.388	0.66 (0.26–1.70)
*MTOR*	rs2295080	Additive	0.049	0.280	0.84 (0.62–1.15)		0.098	0.405	0.81 (0.50–1.33)
*PGF*	rs8185	Dominant	0.395	—			0.068	0.129	1.99 (0.84–4.74)
*PIK3CA*	rs7651265	Dominant	0.031	0.025	0.43 (0.19–0.96)		0.139	—	
*PIK3CA*	rs7640662	Dominant	0.084	0.120	1.64 (0.90–3.00)		0.304	—	
*PTGS2*	rs5275	Recessive	0.004	0.053	3.03 (1.10–8.33)		0.005	0.010*	5.22 (1.70–15.98)
*RET*	rs1799939	Additive	0.037	0.150	0.79 (0.56–1.10)		0.078	0.284	0.74 (0.42–1.30)
*RICTOR*	rs2043112	Recessive	0.097	0.140	1.77 (0.79–3.97)		0.059	0.058	3.40 (0.78–14.95)
*TNF*	rs361525	Additive	0.911	—			0.08	0.820	0.87 (0.26–2.88)
*VEGFA*	rs699947	Dominant	0.109	—			0.046	0.148	0.50 (0.20–1.27)
*VEGFA*	rs25648	Dominant	0.189	—			0.015	0.159	0.43 (0.12–1.53)

Abbreviations: CI, confidence interval; HR, hazard ratio; rs, reference SNP; SNP, single nucleotide polymorphism

Multivariate analysis includes MSKCC risk groups and prior nephrectomy as covariates for progression-free survival and, histology, MSKCC risk groups and prior nephrectomy for cancer-specific survival.

*P value remained significant after adjustment for multiplicity using Benjamini-Hochberg method.

**Table 4:** Univariate and multivariate analyses of two-SNPs combination models associated with progression-free survival and cancer-specific survival in patients with metastatic renal cell carcinoma treated with sunitinib.

	Progression-free survival			
	HR (95% CI)	*P* (Univariate)	HR (95% CI)	*P* * (Multivariate)
***PTGS2* & *PIK3CA***				
rs5275 or rs7651265 *vs*. none	2.45 (1.10–5.44)	0.029	2.84 (1.18–6.80)	0.020
rs5275 & rs7651265 *vs*. none	7.83 (2.43–25.19)	0.001	5.44 (1.39–21.32)	0.015

	**Cancer-specific survival**			
	**HR (95% CI)**	***P* (Univariate)**	**HR (95% CI)**	*P* * (Multivariate)
***PTGS2* & *IL4***				
rs5275 or rs2243250 *vs*. none	2.10 (0.88–5.02)	0.094	4.02 (1.52–10.63)	0.005
rs5275 & rs2243250 *vs*. none	5.89 (2.09–16.58)	0.001	7.32 (2.10–25.54)	0.002

Abbreviations: CI, confidence interval; HR, hazard ratio; rs, reference SNP

Multivariate analysis includes MSKCC risk groups and prior nephrectomy as covariates for progression-free survival and, histology, MSKCC risk groups and prior nephrectomy for cancer-specific survival.

*P value remained significant after adjustment for multiplicity using Benjamini-Hochberg method.
