# Supplementary material for: Functional PTGS2 polymorphism-based models as novel predictive markers in metastatic renal cell carcinoma patients receiving first-line sunitinib
Source: Sci Rep. 2017 Jan 24;7:41371. doi: 10.1038/srep41371 (PMC5259767; doi:10.1038/srep41371)
Supplement: Supplementary Information [file srep41371-s1.pdf]

Title

Functional PTGS2 polymorphism-based models as novel predictive markers in metastatic renal cell carcinoma patients receiving first-line sunitinib

Authors

Arancha Cebrián[§1], Teresa Gómez del Pulgar[§1], María José Méndez-Vidal[2], María Luisa Gonzálvez[3], Nuria Lainez[4], Daniel Castellano[5], Iciar García-Carbonero[6], Emilio Esteban[7], Maria Isabel Sáez[8], Rosa Villatoro[9], Cristina Suárez[10], Alfredo Carrato[11], Javier Munárriz-Ferrándiz[12], Laura Basterrechea[13], Mirta García-Alonso[14], José Luis González-Larriba[15], Begoña Perez-Valderrama[16], Josefina Cruz-Jurado[17], Aránzazu González del Alba[18], Fernando Moreno[19], Gaspar Reynés[20], María Rodríguez-Remírez[1], Valentina Boni[21], Ignacio Mahillo-Fernández[1], Yolanda Martin[22], Andrea Viqueira[23], Jesús García-Foncillas[1]

Author´s affiliation

[1] Fundación Jiménez Díaz University Hospital, Madrid, Spain; [2] Reina Sofía University Hospital, Córdoba, Spain; [3] Virgen de la Arrixaca University Hospital, Murcia, Spain; [4] Complejo Hospitalario de Navarra, Pamplona, Spain; [5] 12 de Octubre University Hospital, Madrid, Spain; [6] Virgen de la Salud University Hospital, Toledo, Spain; [7] Central University Hospital of Asturias, Oviedo, Spain; [8] Virgen de la Victoria University Hospital, Málaga, Spain; [9] Hospital Costa del Sol, Marbella, Spain; [10] Vall d'Hebron University Hospital, Barcelona, Spain; [11] Ramon y Cajal University Hospital, Madrid, Spain; [12] Hospital Provincial de Castellón, Castellón, Spain; [13] Hospital de Donostia, San Sebastian, Spain; [14] Insular de Gran Canaria University Hospital, Islas Canarias, Spain; [15] Clinico San Carlos University Hospital, Madrid, Spain; [16] Virgen del Rocío University Hospital, Sevilla, Spain; [17] Canarias University Hospital, Santa Cruz de Tenerife, Islas Canarias, Spain; [18] Son Espases University Hospital, Palma de Mallorca,

Spain; [19] Fuenlabrada University Hospital, Fuenlabrada, Spain; [20] La Fe University Hospital, Valencia, Spain; [21] Center for Applied Medical Research, Pamplona, Spain; [22] Trial Form Support, Madrid, Spain; [23] Pfizer Oncology, Madrid, Spain

[§] These authors have contributed equally to this work

Supplementary Table S1. Results of each polymorphism in univariate analyses for progression-free survival and cancer-specific survival

			**Progression-free survival**		**Cancer-specific survival**	
Gene	SNP	Inheritance model	*P*	HR (95% CI)	*P*	HR (95% CI)
AKT1	rs3803304	Additive	0.039	0.66 (0.43-0.99)	0.103	0.60 (0.31-1.14)
AKT1	rs2498804	Dominant	0.111	0.65 (0.39-1.10)	0.906	0.95 (0.44-2.06)
AKT1	rs2494738	Dominant	0.530	1.25 (0.63-2.50)	0.058	2.37 (1.03-5.46)
AKT1	rs1130214	Additive	0.837	1.05 (0.67-1.63)	0.599	0.84 (0.43-1.63)
AKT2	rs8100018	Additive	0.917	0.98 (0.63-1.52)	0.173	1.53 (0.84-2.80)
AKT2	rs892119	Additive	0.503	1.21 (0.70-2.09)	0.441	1.37 (0.63-3.00)
AKT3	rs12045585	Additive	0.519	1.20 (0.69-2.07)	0.494	1.31 (0.61-2.80)
AKT3	rs2994329	Additive	0.811	1.06 (0.64-1.79)	0.865	1.07 (0.51-2.25)
ARNT	rs2228099	Dominant	0.145	0.66 (0.38-1.14)	0.273	0.65 (0.30-1.39)
CXCL12	rs1801157	Dominant	0.946	0.98 (0.58-1.67)	0.940	1.03 (0.48-2.20)
FGFR2	rs2981582	Additive	0.265	1.21 (0.87-1.68)	0.438	1.20 (0.76-1.90)
FGFR4	rs351855	Additive	0.239	0.78 (0.51-1.19)	0.202	0.66 (0.35-1.27)
FLT3	rs1933437	Additive	0.321	1.25 (0.80-1.94)	0.284	1.40 (0.75-2.62)
FLT4	rs307826	Additive	0.043	1.80 (1.04-3.13)	0.863	1.07 (0.51-2.24)
HIF1A	rs11549465	Dominant	0.277	1.40 (0.77-2.52)	0.410	1.44 (0.62-3.53)
IL1B	rs1143634	Dominant	0.908	0.97 (0.57-1.66)	0.815	1.09 (0.51-2.33)
IL4	rs2243250	Dominant	0.224	1.45 (0.81-2.60)	0.016	2.62 (1.23-5.61)
IL8	rs4073	Dominant	0.352	1.30 (0.74-2.30)	0.806	1.11 (0.49-2.48)
IL10	rs1800896	Dominant	0.772	1.13 (0.49-2.58)	0.262	2.10 (0.50-8.87)
IL10	rs1800872	Dominant	0.459	1.22 (0.72-2.07)	0.815	1.09 (0.51-2.33)
KDR	rs2305948	Dominant	0.171	0.55 (0.22-1.39)	0.789	1.16 (0.40-3.36)
KDR	rs1870377	Dominant	0.762	0.92 (0.54-1.56)	0.085	0.50 (0.22-1.14)
KDR	rs2071559	Additive	0.819	1.04 (0.74-1.46)	0.701	0.91 (0.56-1.47)
KDR	rs7692791	Dominant	0.154	1.57 (0.82-2.98)	0.171	1.99 (0.69-5.77)
KDR	rs1531289	Additive	0.176	0.75 (0.50-1.13)	0.646	0.87 (0.49-1.55)
MTOR	rs11121704	Dominant	0.372	0.79 (0.47-1.33)	0.218	0.62 (0.29-1.34)
MTOR	rs2295080	Additive	0.049	0.68 (0.46-1.01)	0.098	0.62 (0.34-1.11)
MTOR	rs1074078	Additive	0.314	0.83 (0.56-1.20)	0.297	0.75 (0.43-1.30)
NOS3	rs1799983	Dominant	0.551	0.84 (0.48-1.48)	0.330	1.54 (0.62-3.83)
PDGFRA	rs35597368	Dominant	0.759	1.10 (0.60-2.02)	0.965	0.98 (0.39-2.44)
PDGFRA	rs1800813	Additive	0.846	0.96 (0.62-1.49)	0.781	0.91 (0.47-1.77)
PDGFRA	rs1800810	Dominant	0.767	0.92 (0.53-1.60)	0.886	1.06 (0.48-2.32)
PDGFRA	rs1800812	Dominant	0.547	0.85 (0.49-1.46)	0.925	1.04 (0.48-2.24)
PGF	rs8185	Dominant	0.395	1.29 (0.73-2.28)	0.068	2.09 (0.97-4.52)
PIK3CA	rs7651265	Dominant	0.031	0.48 (0.24-0.99)	0.139	0.48 (0.16-1.38)
PIK3CA	rs7640662	Dominant	0.084	1.67 (0.95-2.94)	0.304	1.54 (0.69-3.43)
PIK3CA	rs7621329	Dominant	0.156	0.68 (0.40-1.16)	0.627	0.83 (0.39-1.77)
PIK3CA	rs6443624	Dominant	0.134	0.67 (0.40-1.13)	0.760	0.89 (0.42-1.89)
PIK3CA	rs2699887	Additive	0.627	1.12 (0.70-1.80)	0.728	0.88 (0.44-1.77)

PTEN	rs2299939	Additive	0.813	0.95 (0.59-1.51)	0.846	0.93 (0.46-1.89)
PTEN	rs12569998	Additive	0.903	1.04 (0.56-1.93)	0.765	0.87 (0.35-2.18)
PTEN	rs12357281	Dominant	0.428	0.72 (0.31-1.68)	0.657	0.77 (0.23-2.55)
PTGS2	rs5275	Recessive	0.004	4.10 (1.81-9.29)	0.005	4.56 (1.82-11.48)
RET	rs1799939	Additive	0.037	0.61 (0.37-1.00)	0.078	0.51 (0.23-1.16)
RHEB	rs717775	Additive	0.312	0.82 (0.56-1.21)	0.747	0.91 (0.52-1.59)
RICTOR	rs2043112	Recessive	0.097	1.32 (0.91-1.89)	0.059	1.35 (0.79-2.38)
RPTOR	rs7211818	Dominant	0.540	1.18 (0.70-2.00)	0.512	1.29 (0.60-2.76)
RPTOR	rs11653499	Additive	0.580	0.90 (0.63-1.30)	0.675	0.90 (0.54-1.50)
RPTOR	rs7212142	Additive	0.215	0.78 (0.53-1.15)	0.889	0.96 (0.55-1.67)
RPTOR	rs9674559	Dominant	0.784	1.08 (0.63-1.84)	0.588	1.24 (0.58-2.64)
TGFB1	rs1800469	Additive	0.436	0.85 (0.56-1.29)	0.488	0.81 (0.44-1.48)
TGFBR1	rs868	Dominant	0.457	1.23 (0.72-2.09)	0.699	0.85 (0.38-1.91)
TNF	rs1800629	Additive	0.509	0.79 (0.38-1.64)	0.438	0.66 (0.22-2.03)
TNF	rs361525	Additive	0.911	0.98 (0.69-1.40)	0.080	0.54 (0.23-1.24)
TNF	rs1799724	Dominant	0.306	0.69 (0.32-1.46)	0.954	1.03 (0.39-2.72)
TSC2	rs2073636	Additive	0.974	1.01 (0.68-1.48)	0.554	1.18 (0.68-2.04)
TSC2	rs8063461	Additive	0.923	1.02 (0.68-1.53)	0.667	1.14 (0.63-2.04)
VEGFA	rs2010963	Dominant	0.751	1.09 (0.63-1.89)	0.514	1.29 (0.59-2.83)
VEGFA	rs1570360	Additive	0.618	0.90 (0.61-1.35)	0.735	0.91 (0.52-1.59)
VEGFA	rs699947	Dominant	0.109	0.64 (0.37-1.09)	0.046	0.46 (0.21-0.97)
VEGFA	rs3025039	Additive	0.456	1.23 (0.72-2.07)	0.383	1.39 (0.68-2.84)
VEGFA	rs25648	Dominant	0.188	0.67 (0.37-1.24)	0.015	0.28 (0.08-0.93)
VEGFA	rs2146323	Dominant	0.198	0.71 (0.42-1.20)	0.268	0.65 (0.30-1.40)

Abbreviations: CI, confidence interval; HR, hazard ratio; rs, reference SNP; SNP, single nucleotide polymorphism

Supplementary Table S2. Results of clinical variables in univariate analyses for progression-free survival and cancer-specific survival

	**Progression-free survival**		**Cancer-specific survival**	
Clinical variable	HR (95%CI)	*P*	HR (95%CI)	*P*
Age	1.00 (0.98-1.02)	0.790	1.02 (0.99-1.05)	0.225
Gender *(Female vs. male)*	1.39 (0.80-2.40)	0.253	1.66 (0.78-3.58)	0.197
Prior nephrectomy *(Yes vs. No)*	0.56 (0.30-1.02)	0.073	0.41 (0.18-0.95)	0.051
MSKCC score *(Poor vs. intermediate)*	2.25 (1.21-4.16)	0.014	2.68 (1.18-6.10)	0.024
Number of metastatic sites *(≥2 vs. <2)*	0.90 (0.54-1.52)	0.699	0.96 (0.45-2.06)	0.926
Histology *(Other vs. clear cell)*	1.75 (0.91-3.36)	0.110	2.30 (0.95-5.57)	0.083
Platelets *(>400000 vs. <400000)*	1.55 (0.82-2.94)	0.199	2.06 (0.87-4.87)	0.125
ECOG *(1-2 vs. 0)*	1.46 (0.61-3.48)	0.383		--

Abbreviations: CI, confidence interval; ECOG, Eastern Cooperative Oncology Group; HR, hazard ratio; MSKCC, Memorial Sloan-Kettering Cancer Center

Supplementary Table S3. Results of AUC for the individual SNPs and their combinations

	**Genetic marker**	**AUC**	**CI**
**PFS**	rs5275	0.595	0.413-0.776
	rs7651265	0.664	0.502-0.826
	rs5275& rs7651265	0.702	0.551-0.853
**CSS**	rs5275	0.649	0.478-0.821
	rs2243250	0.649	0.514-0.785
	rs5275& rs2243250	0.671	0.536-0.807

Abbreviations: AUC, area under the curve; CI, confidence interval; CSS, cancer-specific survival; PFS, progression-free survival; rs, reference SNP

Supplementary Table S4. Univariate and multivariate analyses of *PTGS2* & *FLT4* and *FLT4* & *PIK3CA* combinations associated with progression-free survival in patients with metastatic renal cell carcinoma treated with sunitinib

	**Progression-free survival**			
	HR (95%CI)	*P* (Univariate)	HR (95%CI)	*P* (Multivariate)
PTGS2* & *FLT4				
rs5275 or rs307826 *vs*. none	1.58 (0.87-2.87)	0.135	1.70 (0.89-3.26)	0.135
rs5275 & rs307826 *vs*. None	17.25 (3.47-85.62)	0.0005	23.24 (4.02-134.34)	0.0004
FLT4* & *PIK3CA				
rs307826 or rs7651265 vs. none	1.92 (0.84-4.34)	0.121	2.01 (0.76-5.27)	0.158
rs307826 & rs7651265 vs. none	2.80 (1.11-7.02)	0.029	4.29 (1.50-12.23)	0.006

Abbreviations: CI, confidence interval; HR, hazard ratio; rs, reference SNP

Multivariate analysis includes MSKCC risk groups and prior nephrectomy as covariates for progression-free survival
